# Anti-plasmodial activity of *Dicoma tomentosa* (Asteraceae) and identification of urospermal A-15-O-acetate as the main active compound

**DOI:** 10.1186/1475-2875-11-289

**Published:** 2012-08-21

**Authors:** Olivia Jansen, Monique Tits, Luc Angenot, Jean-Pierre Nicolas, Patrick De Mol, Jean-Baptiste Nikiema, Michel Frédérich

**Affiliations:** 1 Laboratoire de Pharmacognosie, Centre Interfacultaire de Recherche du Médicament (CIRM), Université de Liège, Av. de I’Hôpital 1, CHU-B36, B-4000, Liège, Belgium; 2 Association Jardins du Monde, 15, rue St Michel, 29190, Brasparts, France; 3 Laboratoire de Microbiologie médicale, Université de Liège, Av. de I’Hôpital 1, B23, B-4000, Liège, Belgium; 4 Unité de Formation et de Recherche en Sciences de la Santé, Université de Ouagadougou, 03 BP 7021, Ouagadougou 03, Burkina Faso

**Keywords:** Antiplasmodial, Asteraceae, Melampolide, Burkina Faso, *Dicoma tomentosa*, Natural compound

## Abstract

**Background:**

Natural products could play an important role in the challenge to discover new anti-malarial drugs. In a previous study, *Dicoma tomentosa* (Asteraceae) was selected for its promising anti-plasmodial activity after a preliminary screening of several plants traditionally used in Burkina Faso to treat malaria. The aim of the present study was to further investigate the anti-plasmodial properties of this plant and to isolate the active anti-plasmodial compounds.

**Methods:**

Eight crude extracts obtained from *D. tomentosa* whole plant were tested *in vitro* against two *Plasmodium falciparum* strains (3D7 and W2) using the p-LDH assay (colorimetric method). The Peters’ four-days suppressive test model (*Plasmodium berghei-*infected mice) was used to evaluate the *in vivo* anti-plasmodial activity. An *in vitro* bioguided fractionation was undertaken on a dichloromethane extract, using preparative HPLC and TLC techniques. The identity of the pure compound was assessed using UV, MS and NMR spectroscopic analysis. *In vitro* cytotoxicity against WI38 human fibroblasts (WST-1 assay) and haemolytic activity were also evaluated for extracts and pure compounds in order to check selectivity.

**Results:**

The best *in vitro* anti-plasmodial results were obtained with the dichloromethane, diethylether, ethylacetate and methanol extracts, which exhibited a high activity (IC_50_ ≤ 5 μg/ml). Hot water and hydroethanolic extracts also showed a good activity (IC_50_ ≤ 15 μg/ml), which confirmed the traditional use and the promising anti-malarial potential of the plant. The activity was also confirmed *in vivo* for all tested extracts. However, most of the active extracts also exhibited cytotoxic activity, but no extract was found to display any haemolytic activity. The bioguided fractionation process allowed to isolate and identify a sesquiterpene lactone (urospermal A-15-O-acetate) as the major anti-plasmodial compound of the plant (IC_50_ < 1 μg/ml against both 3D7 and W2 strains). This was also found to be the main cytotoxic compound (SI = 3.3). While this melampolide has already been described in the plant, this paper is the first report on the biological properties of this compound.

**Conclusions:**

The present study highlighted the very promising anti-plasmodial activity of *D. tomentosa* and enabled to identify its main active compound, urospermal A-15-O-acetate. The high anti-plasmodial activity of this compound merits further study about its anti-plasmodial mechanism of action. The active extracts of *D. tomentosa*, as well as urospermal A 15-O-acetate, displayed only a moderate selectivity, and further studies are needed to assess the safety of the use of the plant by the local population.

## Background

Chemotherapeutic treatment of malaria has evolved in the last ten years due to the spread of multi-resistant *Plasmodium falciparum* strains. The World Health Organization (WHO) currently promotes artemisinin-based combination therapy (ACT) as the reference medicine for health care management of uncomplicated falciparum malaria in order to reduce the risk of resistance [1]. However, some signs of resistance to artemisinin have recently been detected in Asia [2-4], representing an enormous threat for the control of the disease. The discovery of new anti-malarial drugs is urgently needed and natural products could play an important role in this new challenge.

The plant kingdom has been and remains a good source of pharmacologically active compounds and especially anti-plasmodial agents, as attested by quinine, isolated from *Cinchona sp.,* and artemisinin extracted from *Artemisia annua*. Besides these well-known examples, various new anti-plasmodial natural compounds are frequently being discovered, as reviewed by several authors in recent years [5-9].

WHO estimates that up to 80% of the world's population relies on traditional medicinal products for some aspects of primary health care [10,11]. Many people living in developing countries do not have access to modern therapeutics, such as ACT, to treat malaria because of financial, socio-economical, geographical and/or cultural reasons, and they use plants, often in combination, for the health care management of malaria.

The search for new anti-malarial natural products, following an ethnopharmacological strategy (based on traditional knowledge of plants), has led to interesting results, as reviewed and commented on by Willcox *et al.*[12,13].

Moreover, there is now evidence that some whole plant extracts can be more active than single compounds, as a result of synergy and positive interactions between different constituents in the extracts, compared to a single product [14,15].

In this context, the pharmacological and phytochemical study of plants from traditional pharmacopoeias can be of interest, not only in discovering new anti-malarial “lead compounds”, but also in valorizing local vegetal species whose efficacy and safety has been demonstrated in laboratory and clinical investigations [16].

Better knowledge of plants from traditional pharmacopoeias and local valorization of traditional remedies in improved traditional medicine (ITM) could lead to effective, standardized, available and affordable therapeutics for the management of malaria by local populations, when modern drugs are unavailable [17].

Validated anti-malarial phytomedicines formulated from traditional medicines have been reported in recent years. Some are government approved in different countries, e g, *Argemone mexicana* in Mali, whose anti-malarial activity has been confirmed in clinical trials [18], and “Saye” in Burkina Faso, a combination of three plants, used for several years as a curative anti-malarial and recently studied for its benefits in the prophylaxis of malaria [19].

In a previous study dealing with the screening of several plants used in Burkina Faso in the traditional treatment of malaria, *Dicoma tomentosa* was selected for its promising anti-plasmodial activity [20].

*Dicoma tomentosa* is a plant of the Asteraceae family growing in Asia and tropical Africa. It is an uncommon species, mainly found in the Sahelo-Sudanian area. Called “Gômtigdà” in the local language (Mooré), the decoction of the whole plant is traditionally used in Burkina Faso to treat malaria in adults and children, particularly malaria with spleen and liver “inflammation” [21]. The plant is also known for its benefits against cough and in postnatal care to “wash the belly”, but it is not recommended for pregnant women because it is known to cause abortion.

The plant has been described as containing several sesquiterpene lactones [22-24], triterpenes and sterols [25,26] as well as flavonoids [27-30], but has never been studied for any biological or pharmacological properties.

The aim of the present study was to further evaluate the anti-plasmodial potential of *D. tomentosa* using *in vitro* and *in vivo* models. *In vitro* cytotoxic and haemolytic properties were also studied in order to check the selectivity of the plant and thereby to appreciate its safety of use.

Finally, a bioguided fractionation was undertaken to complete the study. This phytochemical analysis led to the isolation and identification of the major active compound.

## Methods

### Plant material

*Dicoma tomentosa* whole plant (400 g) was collected in Poun, province of Sanguié, Burkina Faso in October 2008. The sample was authenticated by the Herbarium of the National Botanic Garden of Belgium, at Meise (Belgium). A voucher specimen was deposited in the same Herbarium under the number BR0000005088959. The plant material was washed and dried in a ventilated room (30°C) and then ground to a powder.

### Preparation of crude extracts

Eight crude extracts were prepared using eight different solvents: petroleum ether, hexane, dichloromethane, diethylether, ethylacetate, methanol, ethanol/water (50%, v/v) and hot water. For each solvent, 5 g of dried plant powder were macerated with 50 ml of solvent, while being shaken for 30 minutes with a magnetic stirrer. This step was repeated twice. The preparations were filtered and evaporated under reduced pressure.

For the aqueous crude extract, we prepared a decoction of 5 g dried plant powder in 150 ml distilled water for 90 min in order to approximate the traditional preparation method. The preparation was then filtered and freeze dried.

### *In vitro* anti-plasmodial assays

#### Culture

Continuous cultures of *P. falciparum,* chloroquine-sensitive (3D7) and chloroquine-resistant (W2) strains were maintained as described by Frédérich *et al.*[31]. Both strains were obtained from Prof Grellier (Museum National d’Histoire Naturelle, Paris, France).

#### Assay

The *in vitro* anti-plasmodial assay reproduces the erythrocytic development stage of the parasite and was performed as previously described [20]. Each of the eight crude extracts was first dissolved in DMSO (Sigma) at a concentration of 10 mg/ml (5 mg/ml for pure compound). *Plasmodium falciparum c*ulture was placed in contact with a set of eight two-fold dilutions in the medium of each extract on two columns of a 96-well microplate for 48 h (final concentrations ranging from 0.8 (0.4) to 100 (50) μg/ml and final DMSO concentration ≤1%, each condition in duplicate). Parasite growth was estimated by colorimetric revelation and measurement of absorbance at 630 nm with a multiwell scanner (Stat Fax 2100, Awareness Technology Inc). The test is based on the evaluation of the plasmodial lactate dehydrogenase (pLDH) activity and was performed according to the method of Makler *et al.*[32] and as described previously by Kenmogne *et al.*[33]. Artemisinin (Sigma–Aldrich) and chloroquine (Sigma–Aldrich) were used as positive standards, and infected and uninfected erythrocytes were added as positive and negative controls, respectively. The inhibition of growth was calculated by comparison with the infected non-treated erythrocytes (=100% growth). IC_50_ values, indicating the concentration of the drug needed to obtain 50% inhibition of parasite growth, were calculated by linear regression from a set of eight concentrations tested for each extract/pure compound. Each sample was tested three times (3 independent assays, n = 3) for each plasmodial strain.

In line with WHO guidelines and basic criteria for antiparasitic drug discovery [34,35], activities of extracts were classified into four classes according to their IC_50_ values: high activity (IC_50_ ≤5 μg/ml); promising activity (5 μg/ml < IC_50_ ≤15 μg/ml); moderate activity (15 μg/ml < IC_50_ ≤50 μg/ml); weak activity (IC_50_ >50 μg/ml). A pure compound is defined as highly active when its IC_50_ ≤1 μg/ml.

### *In vitro* cytotoxicity assays

#### Culture

WI-38 normal human fœtal lung fibroblasts were maintained in continuous culture in DMEM medium (Bio Whittaker) in a humid atmosphere at 37°C and 5.5% CO_2_. Each medium was supplemented with 10% heat-inactivated fœtal bovine serum (Bio Whittaker), 1% L-glutamine (200 mM) (Bio Whittaker) and antibiotics: penicillin (100 UI/ml) – streptomycin (100 μg/ml) (Pen-strep®, Bio Whittaker).

#### Assays

Each of the eight crude extracts was first dissolved in DMSO (Sigma) to a concentration of 10 mg/ml (5 mg/ml for pure compound). For the assays, 96-well tissue culture microplates (Micro Test-96®, Falcon, Becton-Dickinson) were seeded with 200 μl medium containing 8,000 cells in suspension.

After 24 h incubation, cells were treated with six dilutions at a final concentration of crude extracts in culture medium of 1, 5, 10, 25, 50 and 100 μg/ml (half concentration for pure compounds) and a final DMSO concentration ≤1%. After 48 h incubation, cell viability was determined by adding WST-1 (Roche Biomolecular) tetrazolium salt as a cytotoxicity indicator and by reading absorbance at 450 nm with a scanning multiwell spectrophotometer (Stat Fax 2100, Awareness Technology Inc) after about a one-hour wait. Tetrazolium salts are cleaved to formazan dye by cellular enzymes. The absorbance directly correlates to the viable cell number. Each condition was reproduced in triplicate and each set of tests was performed twice. Camptothecin (Sigma-Aldrich) was used as a positive cytotoxic control. The inhibition of growth was calculated by comparison with the negative control (non-treated cells). IC_50_ values, indicating the concentration of the drug needed to obtain 50% inhibition of cell growth, were calculated by linear regression from a set of six concentrations tested for each extract.

#### Selectivity index (SI)

The SI value allows the evaluation of the selective activity of the extracts/pure compound against the parasite compared to its toxicity for human cells. The SI value is calculated as the ratio between cytotoxic IC_50_ values and 3D7 or W2 parasitic IC_50_ values.

### *In vitro* haemolysis assays

Haemolysis assays were conducted with the eight crude extracts and isolated pure compound, according to a previously described procedure [36]. Briefly, red blood cells suspensions (10% in PBS (v/v)) were incubated under agitation at room temperature for one hour with extract or pure compound solutions (final concentration = 100 μg/ml and DMSO <1%). The mixtures were then centrifuged at room temperature for 5 min at 10,000 × g and the absorbance (OD) of the supernatants was measured at 550 nm with a microplate reader (Stat Fax 2100, Awareness Technology Inc). The crude extracts/pure compound were tested in triplicate at a final concentration of 100 μg/ml. Triton X-100 1% (v/v) was used as a positive control (100% red blood cell lysis) and PBS as a negative control (0% red blood cell lysis). The red blood cell lysis percentage (H) was determined as follows: H = (OD550nm sample − OD550nm PBS)/(OD550nm Triton X-100 1% − OD550nm PBS). The results were expressed as means ± SD with n = 3.

### *In vivo* anti-plasmodial assays

The present study was approved by the Ethical Committee for the use of laboratory animals at the University of Liège (no. 721) and was designed according to the internationally recognized guidelines. Methanol, hydroethanolic and aqueous extracts of *D. tomentosa* were tested using a test protocol based on the four-day suppressive test of Peters [35]. Female Swiss mice (10 weeks of age, 25 ± 2 g), obtained from Charles River Laboratories (Brussels), were infected by the rodent parasite *Plasmodium berghei* NK173, following the protocol described in Frederich *et al.*[37]. Groups of five mice were formed randomly. The treatment doses (100 mg/kg ip or 300 mg/kg po, dissolved in 7% Tween 80 and 3% ethanol) were given intraperitoneally (methanol and hydroethanolic extracts) or orally (hydroethanolic and aqueous extracts), four hours after infection (day 0). The treatment was repeated once daily for the next three days (on days 1, 2 and 3 post-infection). On day 4 and day 7, thin blood smears were made from mouse-tail blood and were stained with Giemsa. Slides were inspected under the microscope and parasitaemia was determined by counting at least 500 erythrocytes. Mice were controlled for their mortality for two weeks. Chloroquine diphosphate salt at 4 mg/kg (ip) was used as positive control and a methanol extract of the bark of *Cinchona succirubra* at 200 mg/kg (ip) was used as “positive plant extract control” due to the known presence of quinine in this *Cinchona* bark extract. This was used as a supplemental positive control to observe the profile of inhibition with a plant extract containing a known active ingredient. The vehicle solution (7% Tween 80 and 3% ethanol) was used as a negative control.

Inhibition of *Plasmodium* growth (%) was calculated by comparison of the parasitaemia counted for the test group with the parasitaemia in the negative control group, at days 4 and 7, respectively. Except for extracts tested by the oral route, which were tested once, the experiment was performed twice and the results represent means of inhibition of parasitaemia obtained for both experiments.

### Bioguided fractionation

#### Extraction

60 g of *D. tomentosa* whole plant powder were first percolated with 0.9 l of hexane followed by 1.8 l of dichloromethane to give 1.1 g of hexane extract (E1) and 1.9 g of dichloromethane extract (E2), respectively (yield E2 = 3.1% (w/w)).

#### TLC analysis

Extracts and fractions were analysed by thin layer chromatography on Si60 silica gel plate (Merck) using dichloromethane-methanol (95:5) as the mobile phase. The plates were observed under UV light (254 and 366 nm) and then revealed by spraying of sulphuric vanillin reagent (2% in ethanol (w/v)) and heating at 105°C for 10 min.

#### Preparative HPLC

300 mg of the dichloromethane extract (E2) were dissolved in 11 ml of methanol–water (50:50). The solution was centrifuged, filtered (0.45 μ membrane) and injected into a reversed-phase preparative HPLC system using a Lichrospher RP-18 column (2.5x25 cm; 12–15 μ, Merck packing system) as the stationary phase. The mobile phase consisted of methanol–water (50:50) in isocratic elution with a flow rate of 20 ml/min (Armen pump). Fractions of 10 ml were collected (Büchi fraction collector) and separation was monitored by TLC analysis. Fractions with the same TLC profiles were assembled to finally give 16 fractions, designated F1 to F16. F16 (99.8 mg) was the insoluble part of the extract collected on the filter. F4 (10.2 mg) and F5 (67.3 mg) contained the main compound of the extract, as shown by TLC analysis.

#### Preparative TLC

10.2 mg of F4 were dissolved in 0.5 ml of dichloromethane and deposited on a preparative Si60 TLC plate (1.5 mm x 20 cm x 20 cm). Elution was performed with dichloromethane-methanol (95:5) as the mobile phase. The band corresponding to the pure **compound 1** was scratched off and extracted with a mixture of dichloromethane and methanol (50:50) using a G4 filter. The filtrate was evaporated under reduced pressure to give 8.0 mg of the pure **compound 1** = urospermal-15-O-acetate. The same procedure was applied to 9.7 mg of F5 to give 7.8 mg of **1**.

#### Spectroscopic analysis

The structure of **compound 1** was elucidated using various spectroscopic techniques. NMR spectra (^1^ H, ^13^C, COSY, HMBC, HSQC) were recorded in CDCl_3_ with a Bruker Avance 500 MHz spectrometer equipped with a cryoprobe and using TMS as the internal reference. Mass spectrometry (MS) was carried out using a micromass ESI-Q-TOF II instrument (Waters) using ESI-ionization in positive mode. The UV spectrum was obtained with a methanol solution using a Kontron Uvikon Spectrophotometer. The identity of the purified **compound 1** was assessed by comparison of its NMR and MS data with data from the literature [22] (Additional file 1).

#### Biological assays

The fractions as well as E1 and E2 and **1** were tested against the 3D7 *P. falciparum* strain according to the protocol described above. The cytotoxic and haemolytic properties of **1** were also evaluated, as well as its anti-plasmodial activity against the W2 chloroquino-resistant strain. We used the same method as described for the crude extracts.

## Results and discussion

### *In vitro* and *in vivo* anti-plasmodial activity of crude extracts

All eight tested extracts were found to be active against both chloroquine-sensitive 3D7 and -resistant W2 strains of *P. falciparum* (IC_50_ < 50 μg/ml). IC_50_ values obtained with crude extracts are detailed in Table 1, as well as extraction yields from plants (w/w). Dichloromethane, diethylether, ethylacetate, and methanol extracts were found to be highly active with IC_50_ ≤ 5 μg/ml, while hydroethanolic and hot water extracts displayed a promising activity (IC_50_ ≤ 15 μg/ml). Petroleum ether and hexane extracts showed a lower level of activity.

**Table 1:** ***In vitro *anti-plasmodial, cytotoxic and haemolytic activity of crude extracts and pure compound (1) obtained from *Dicoma tomentosa (n = 3)***

**Pure compound**	**Yield (in the plant)**	**IC**_**50**_**3D7 μg/ml (μM)**	**IC**_**50**_**W2 μg/ml (μM)**	**IC**_**50**_**WI38 μg/ml (μM)**	**SI 3D7**	**SI W2**	**Haemolysis (%)**
Compound 1 = urospermal-15-O-acetate (MM = 320.34)	0.64%	0.92 ± 0.05 (= 2.87 ± 0.16)	0.77 ± 0.25 (= 2.41 ± 0.78)	3.03 ± 0.18 ( = 9.47 ± 0.56)	3.3	3.9	< 1%
**Extracts**	**Yield (%)**	**IC**_**50**_**3D7 (μg/ml)**	**IC**_**50**_**W2 (μg/ml)**	**IC**_**50**_**WI38 (μg/ml)**	**SI 3D7**	**SI W2**	**Haemolysis (%)**
Petroleum ether	1.8%	23.2 ± 3.1	21.2 ± 2.2	22.0 ± 0.6	0.9	1.0	< 1%
Hexane	1.7%	18.7 ± 2.9	17.7 ± 1.2	32.9 ± 4.0	1.8	1.9	< 1%
Dichloromethane	5.4%	3.4 ± 1.2	1.9 ± 0.2	11.5 ± 0.4	3.3	6.1	< 1%
Diethylether	3.4%	3.9 ± 0.6	4.8 ± 0.7	6.5 ± 1.4	1.6	1.3	< 1%
Ethylacetate	4.1%	4.4 ± 1.1	4.6 ± 0.3	5.9 ± 0.6	1.3	1.3	< 1%
Methanol	11.1%	5.8 ± 2.0	3.0 ± 0.5	19.9 ± 5.4	3.5	6.7	< 1%
Ethanol 50% (v/v)	16.2%	12.9 ± 3.0	9.7 ± 1.1	28.4 ± 2.9	2.2	2.9	< 1%
Hot water	20.3%	12.8 ± 2.4	6.7 ± 1.0	47.2 ± 2.9	3.7	7.0	< 1%

SI = selectivity index = IC_50(WI38)_/IC_50(3D7 or W2, respectively)_

Standard drugs: artemisinin (3D7): IC_50_ = 0.0067 ± 0.0018 μg/ml; artemisinin (W2): IC_50_ = 0.0034 ± 0.0009 μg/ml; chloroquine (3D7): IC_50_ = 0.016 ± 0.004 μg/ml; chloroquine (W2): IC_50_ = 0.35 ± 0.08 μg/ml; camptothecin (WI-38): IC_50_ = 0.029 ± 0.011 μg/ml.

The levels of activity observed with the dichloromethane, methanol and hot water extracts were about twice as high as the anti-plasmodial IC_50_ values obtained for the same extracts in the previous study. That previous study was conducted with a batch of *D. tomentosa* also collected in Burkina Faso, at the same time of year but in 2007 and in a different area (and with samples from stock maintained in less adequate storage conditions) [20]. The anti-plasmodial activity obtained with this new batch was found to be in the same range as can be observed with *Artemisia annua* extracts [38]. The anti-plasmodial activity of the hot water extract supports the traditional use of this plant to treat malaria and is an interesting result when considering further prospects for its local valorization.

**Figure 1:**
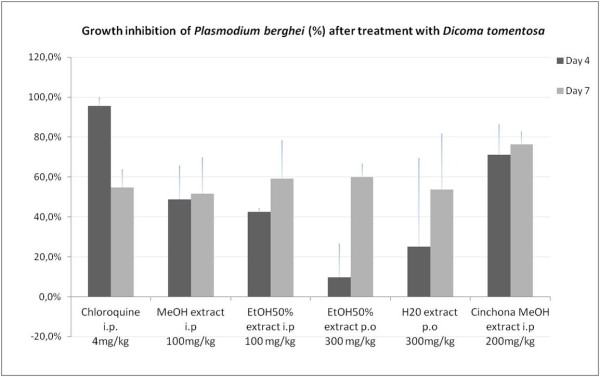
***In vivo *anti-plasmodial activity is expressed by the inhibition of the growth of *Plasmodium berghei *in infected mice treated with *Dicoma tomentosa *extracts compared to a non-treated negative control group of mice (growth = 100%).** Standard drug = chloroquine (4 mg/kg, ip) and *Cinchona succirubra* stem bark methanol extract (200 mg/kg, ip) MeOH = methanol; EtOH50% = Ethanol/Water 50% (v/v); H20 = Hot water.

*In vivo* experiments with methanol, hydroethanolic and aqueous extracts of *D. tomentosa* whole plant confirmed the anti-plasmodial activity of this Asteraceae. Both treatments given intraperitoneally (methanol and hydroethanolic extracts, 100 mg/kg) displayed an inhibition of parasitaemia in the range of 40-60% at days 4 and 7. Unfortunately, both extracts were found to be toxic when the dose was increased to 200 mg/kg/day, i.p. (all the mice died before day 4). Both treatments given by oral route (hydroethanolic and hot water extracts, 300 mg/kg) showed a much lower activity at day 4, but at day 7 the inhibition of *Plasmodium* growth was in the same range as for the extracts given intraperitoneally. Detailed results are presented in Figure 1.

The inhibition of parasitaemia was thus maintained (for ip-tested extracts and *Cinchona* control extract) or improved (for po-tested extracts) between days 4 and 7, in contrast with chloroquine, for which the activity was high at day 4 but clearly decreased at day 7. The prolonged activity obtained with the plant extracts against the parasite indicates either a long half-life of the active compounds in the extract, or the implication of metabolization in the activity with production of active metabolites. That (as well as the gastrointestinal resorption process) could explain the delayed effect observed with oral treatments.

### Cytotoxic and haemolytic activity of crude extracts

Additional *in vitro* studies concerning the haemolytic potential and the cytotoxic activity on normal human fibroblasts were also carried out in order to check the anti-plasmodial selectivity of *D. tomentosa*. Results are detailed in Table 1.

No extract was found to exhibit significant red blood cells lysis activity with a percentage of haemolysis <1% for all tested extracts (conc = 100 μg/ml). This indicates that anti-plasmodial activity is not correlated with haemolysis of red blood cells but with a real action against the parasite.

However, all eight extracts were found to be quite cytotoxic on WI38 cells (IC_50_ values from 5.9 to 47.2 μg/ml) and showed a low to moderate selectivity (SI from 0.9 to 7.0, depending on the extract and the plasmodial strain).

### Bioguided fractionation: isolation and identification of the major active compound

A bioguided fractionation was carried out in order to identify the compound(s) responsible for the anti-plasmodial activity.

Preliminary TLC analysis of the CH_2_Cl_2_ crude extract revealed the presence of some major terpenic compounds in the plant, especially one grey spot (Rf ~0.65), using the sulphuric vanillin reagent. The intensity of this grey spot, named **“compound 1”**, seemed to be correlated with the *in vitro* anti-plasmodial activity of the different tested extracts (Additional file 2).

For the bioguided fractionation and depending on the results obtained with the eight crude extracts, powdered plant was first extracted by hexane (E1), followed by dichloromethane (E2 = “defatted extract” – yield = 3.1%).

E2 was submitted to preparative reversed-phase HPLC to give 16 fractions (F1-F16). The activity of the extracts and fractions was evaluated against *P. falciparum* 3D7 strain. IC_50_ values of E1 and E2 were 36.9 and 2.0 μg/ml, respectively. E2 containing **compound 1** as the major compound was found to be even more active than the crude dichloromethane extract, while E1, found to contain the other major terpenes in TLC analysis, was much less active.

All tested fractions obtained from E2 were found to possess high or promising anti-plasmodial activity, with IC_50_ values ranging from 0.95 μg/ml (F4 and F5) to 6.9 μg/ml (F16).

The significant activity detected in several fractions with different TLC profiles means that several compounds can be said to contribute to the overall *in vitro* anti-plasmodial properties of *D. tomentosa* dichloromethane extract. However, major **compound 1** - the grey spot - was the main constituent of the two most active fractions F4 and F5 (respectively 10.2 mg and 67.3 mg = 25.84% E2 (w/w)) and this compound could therefore be considered as the major active compound in the plant.

**Figure 2:**
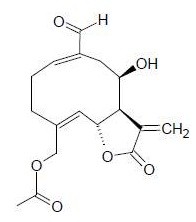
urospermal A-15-O-acetate (MM = 320.34).

**Compound 1**, a terpene, was finally isolated as a pure compound after an additional preparative TLC step on F4 and F5 and was submitted to UV, MS/MS and NMR analysis. **Compound 1** was identified as urospermal A-15-O-acetate, a melampolide-type sesquiterpene lactone (Figure 2). Its identity was confirmed by comparison of its spectral profile with spectral data available for known compounds in *D. tomentosa*[22]. Melampolides are 10-membered ring sesquiterpene lactones. They can be considered a subgroup of germacranolides but with a 1,10 cis-double bond configuration. 

Yield of urospermal A-15-O-acetate was estimated to 20.7% (w/w) of the dichloromethane extract (E2), corresponding to 0.64% (w/w) of the dried plant.

### Anti-plasmodial, cytotoxic and haemolytic properties of urospermal A-15-O-acetate

Purified **compound 1** was also tested for its anti-plasmodial, cytotoxic and haemolytic properties. Results are presented in Table 1. Urospermal A-15-O-acetate was found to display a very strong anti-plasmodial activity with IC_50_ <1 μg/ml against both chloroquine-sensitive and -resistant *P. falciparum* strains. The contribution of this active compound to the activity of the dichloromethane extract (E2) can be estimated to 62.1%, according to the method described by Deharo and Ginsburg [15]. This confirmed that urospermal A-15-O-acetate could be considered as the main active compound of the plant. It is likely that other minor closely related sesquiterpene lactones – such as the other germacranolides and melampolides already described in the plant [22,23] – also displayed additive/synergistic anti-plasmodial activity, as attested by the significant activity detected in almost all the fractions. Products from other phytochemical classes, such as flavonoids (already described in the plant [27-30]) could also play some role in the overall anti-plasmodial effect by several mechanisms, as widely described for *A. annua*[38,39].

No significant haemolytic activity was detected for **1**. However, this sesquiterpene lactone appeared to be cytotoxic with IC_50_ = 3.0 μg/ml on WI38 human fibroblasts. The selectivity index (SI = 3.3) was the same as those obtained with the crude CH_2_Cl_2_ extract, which suggests that this major anti-plasmodial compound is the main cytotoxic product in the plant.

Urospermal A-15-O-acetate has already been described as a major compound of *D. tomentosa* by Bolhmann *et al.*[22] for a batch collected in South Africa. The present study is, however, the first report of the compound’s anti-plasmodial and cytotoxic properties. Moreover, this compound has never been described in any other plant.

Recently, another plant from the same genus, *Dicoma anomala ssp gerrardii* was described for its anti-plasmodial activity [40]. The main active compound in that plant was also a sesquiterpene lactone but of the eudesmanolide-type and represented 0.0013% (w:w) of the dried plant powder. Cytotoxic activity was also detected for this compound in that study.

Many sesquiterpene lactones isolated from Asteraceae have already been described as anti-plasmodial and cytotoxic in the literature, such as (pseudo)guaianolides, eudesmanolides and also germacranolides/melampolides (e g, tagitinin C from *Tithonia diversifolia*[41] and recently acanthospermolide derivatives from *Acanthospermum hispidum*[42]). Structure–activity relationship studies have shown that the α-methylene-γ-lactone moeity (or more widely, at least one potentially reactive α,β-unsaturated bond) is the main element needed for anti-plasmodial or cytotoxic activity of such compounds, and that antiprotozoal activity is significantly correlated with cytotoxicity [43,44]. The low selectivity of such compounds can be explained by the chemical reactivity of the α,β-unsaturated bond, especially towards free thiol groups (e g, cysteine residues in enzymes and transcription factors).

## Conclusions

*Dicoma tomentosa* showed promising anti-plasmodial properties through *in vitro* and *in vivo* tests performed in the present study, and this supports the traditional use of this plant. However, the plant’s lack of selectivity would urge caution in its consumption by the local population. Further studies (e g, regarding genotoxicity, acute/chronic toxicity) are needed to assess the safe use of the plant.

We found that the main active compound of the plant, urospermal A-15-O-acetate, showed a promising anti-plasmodial activity with a low but real selectivity. Its mechanism of action is currently under study. A pharmacomodulation process may need to be undertaken in order to decrease the compound’s toxicity while maintaining (or improving) its activity. On the other hand, its cytotoxic activity could also be investigated in the field of cancerology.

## Abbreviations

WHO:World Health Organization; ACT:Artemisinin combination therapy; ITM:Improved traditional medicine; SI:Selectivity index; CQ:Chloroquine; HPLC:High performance liquid chromatography; TLC:Thin layer chromatography; ip:Intra-peritoneal; po:Per os.

## Competing interests

The authors declare that they have no competing interests.

## Authors’ contributions

OJ carried out the *in vitro* and *in vivo* anti-plasmodial activity assays and the bioguided fractionation as well as the *in vitro* cytotoxic and haemolytic assays. She also analysed the data and drafted the manuscript. MT and LA contributed to data analysis and critically revised the manuscript. JPN participated in plant selection and collection, analysed botanical/ethnomedicinal data and contributed to the revision of the manuscript. PDM and JBN assisted with data analysis and the revision of the manuscript. MF contributed to the design of experiments, helped in *in vivo* anti-plasmodial assay and data analysis and assisted in the correction of the draft manuscript. All the authors read and approved the final manuscript.

## Supplementary Material

Additional file 1 Spectroscopic data of compound 1. Click here for file

Additional file 2 **TLC analysis of *D. tomentosa* extracts tested *in vitro*for antiplasmodial activity.** Click here for file
