# Supplementary material for: Anti-plasmodial activity of Dicoma tomentosa (Asteraceae) and identification of urospermal A-15-O-acetate as the main active compound
Source: Malar J. 2012 Aug 21;11:289. doi: 10.1186/1475-2875-11-289 (PMC3483198; doi:10.1186/1475-2875-11-289)
Supplement: Additional file 1 — Spectroscopic data of compound 1. [file 1475-2875-11-289-S1.docx]

**Spectroscopic data of compound 1**


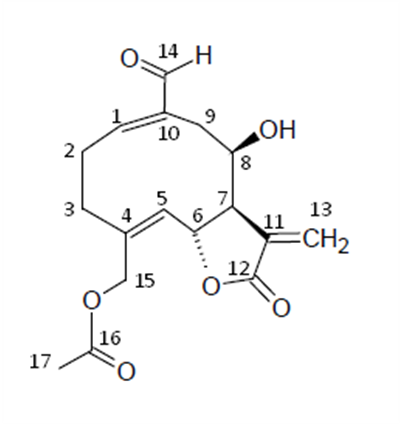


**Common name** : Urospermal A -15-O-acetate

**Molecular formula** : C_17_H_20_O_6_ (MM = 320.3)

**MS** : [*m/z]^+^* = 343.1 [M+Na]*^+^*

**UV** : λ_MAX_ = 229.5 nm

**NMR** : Chemical shifts (δ) in ppm from TMS.

^1^H-NMR spectroscopic data (500MHz, CDCl3):

2.08 (1H, m, H3’); 2.13 (3H, s, H17); 2.37 (1H, m, H2’); 2.48 (1H, m, H7); 2.50 (1H, m, H9’); 2.52 (1H, m, H9); 2.61 (1H, m, H2); 2.67 (1H, m, H3); 3.98 (1H, m, H8); 4.60 (1H, dd - J=10.6Hz, 10.3Hz, H6); 4.71 (1H, d - J=12.8Hz, H15’); 4.84 (1H, d - J=12.8Hz, H15); 5.22 (1H, d-J=10.6Hz, H5); 5.63 (1H, d, OH); 6.33 (1H, m, H13); 6.54 (1H, m, H13’); 6.83 (1H, t, H1); 9.47 (1H,s, H-14).

^13^C-NMR spectroscopic data (500MHz, CDCl3):

20.8 (CH_3_, C-17); 27.7 (CH_2_, C-2); 32.9 (CH_2_,C-3); 33.2 (CH_2_, C-9); 51.7 (CH, C-7); 61.9 (CH_2_, C-15); 70.0 (CH, C-8); 75.5 (CH, C-6); 125.4 (CH_2_, C-13); 129.9 (CH, C-5); 136.4 (C, C-11); 136.6 (C, C-4); 144.4 (C, C-10); 159.2 (CH,C-1);170.0 (C, C-12); 170.5 (C, C-16); 199.3 (C, C-14).
