# Supplementary material for: Anti-plasmodial activity of Dicoma tomentosa (Asteraceae) and identification of urospermal A-15-O-acetate as the main active compound
Source: Malar J. 2012 Aug 21;11:289. doi: 10.1186/1475-2875-11-289 (PMC3483198; doi:10.1186/1475-2875-11-289)
Supplement: Additional file 2 — TLC analysis of D. tomentosa extracts tested in vitrofor antiplasmodial activity. [file 1475-2875-11-289-S2.pdf]

# TLC analysis of *D. tomentosa* extracts tested *in vitro* for antiplasmodial activity

COMPOUND 1

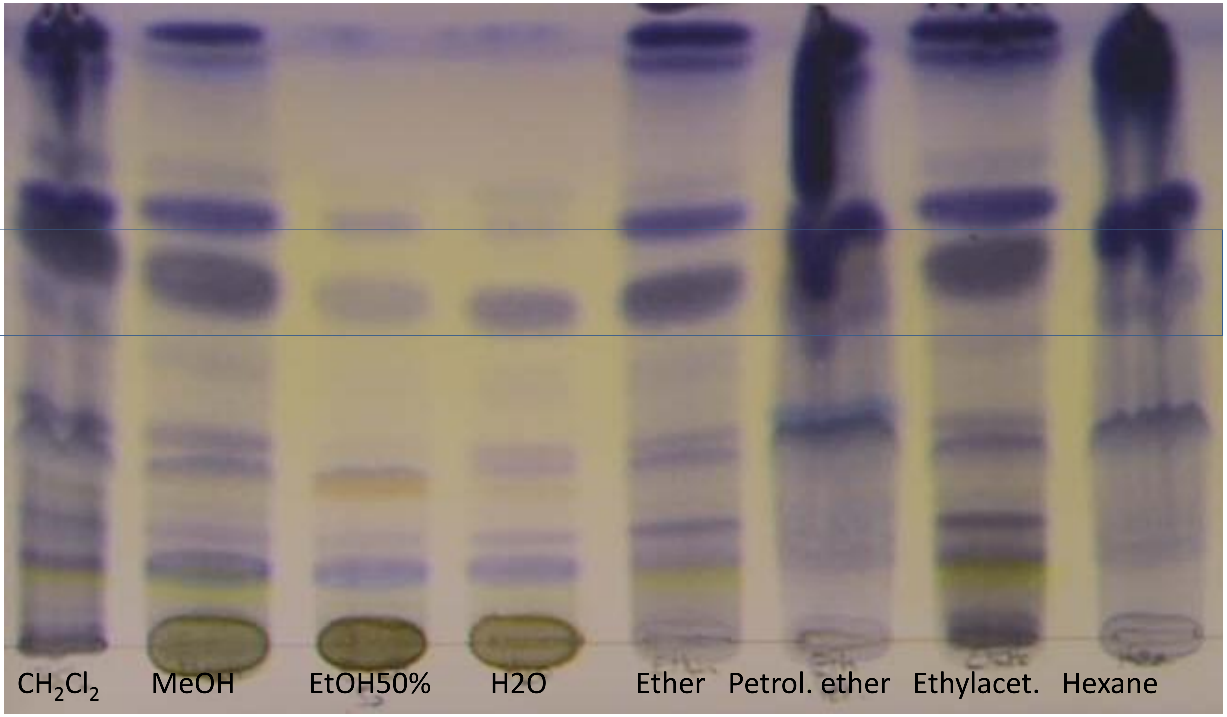


| | | | | | | | | |
|---|---|---|---|---|---|---|---|---|
| $IC_{50}$ | 3.4 | 5.8 | 12.9 | 12.8 | 3.9 | 23.2 | 4.4 | 18.7 |

$IC_{50}$ in µg/ml (*P. falciparum* 3D7)

Mobile phase : $CH_2Cl_2$ - MeOH (95:5)
Revealed using sulphuric vanillin reagent (see Materials and methods)

## **Bioguided fractionation of *D. tomentosa***

$IC_{50}$ in µg/ml (*P. falciparum* 3D7)

Right : TLC

Extracts ($CH_2Cl_2$ total crude extract, E1 and E2 (subsequent hexane and $CH_2Cl_2$ extracts, in this order respectively)

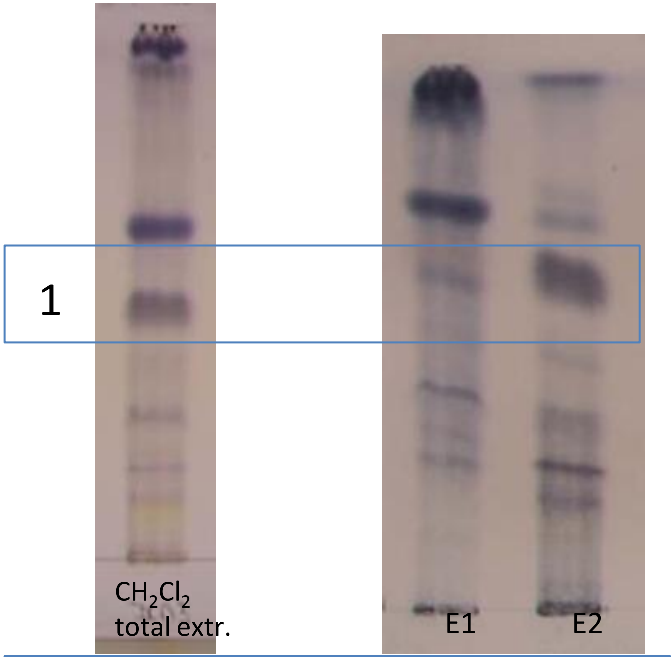


| | | | |
|---|---|---|---|
| $IC_{50}$ | 3.4 | 36.9 | 2.0 |

Below: TLC

Fractions collected from preparative HPLC (E2)

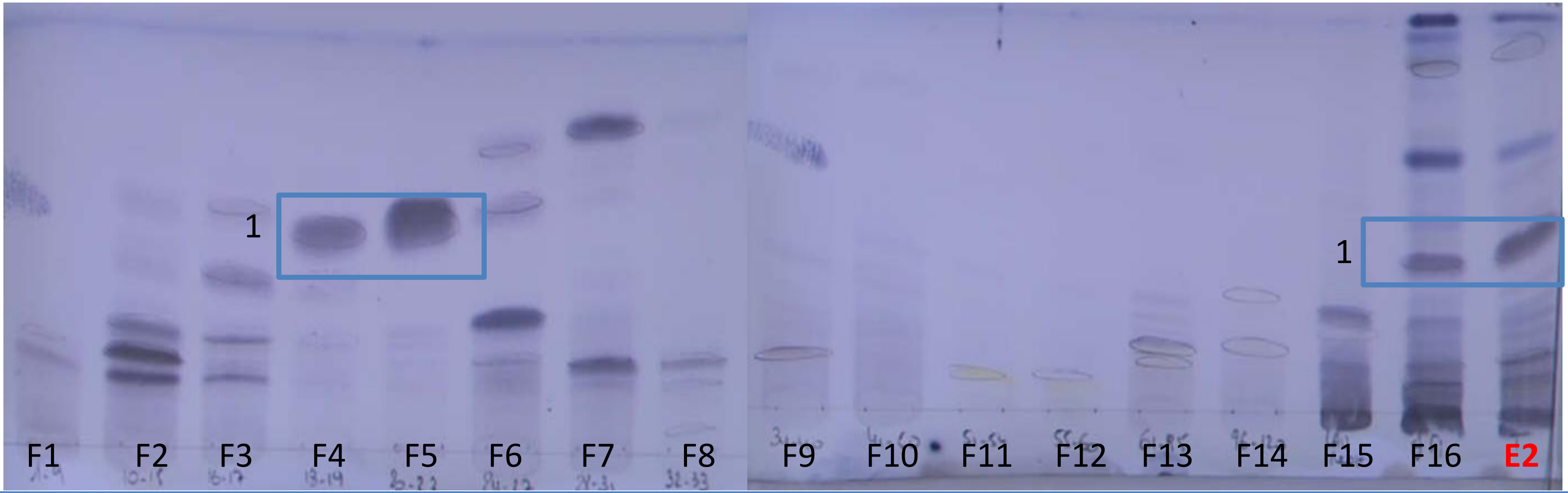


| | | | | | | | | | | | | | | | | | |
|---|---|---|---|---|---|---|---|---|---|---|---|---|---|---|---|---|---|
| $IC_{50}$ | 2.31 | 1.26 | 1.06 | 0.95 | 0.95 | 1.31 | 1.65 | ND | 1.96 | ND | 3.32 | ND | 4.39 | ND | 4.19 | 6.9 | 2.0 |
